# Editorial: the 19th annual *Nucleic Acids Research* web server issue 2021

**DOI:** 10.1093/nar/gkab525

**Published:** 2021-06-25

**Authors:** Dominik Seelow

**Affiliations:** Exploratory Diagnostic Sciences, Berliner Institut für Gesundheitsforschung, Berlin 10117, Germany,Germany; Institut für Medizinische Genetik und Humangenetik, Charité – Universitätsmedizin Berlin, corporate member of Freie Universität Berlin and Humboldt-Universität zu Berlin, Berlin 13353, Germany Germany

The 2021 edition is the 19th Web Server Issue. For this issue, we received 297 proposals. The authors of 110 (37%) proposals were invited to submit a manuscript. 89 (81%) of the submissions were accepted, corresponding to 30% of initial proposals being successful.

As in the previous years, submitted proposals were first checked for their general suitability for the Web Server Issue. As this special issue is aimed at web-based implementations of peer-reviewed methods, web servers that heavily depend on new and unpublished methods were not considered for publication. We also maintain an embargo of two years before re-publication of a web server, including manuscripts in other journals. Updates of existing web servers must provide a clear benefit to potential users.

Last but not least, the web server must be of general interest for our readership. This usually excludes tools aimed at individual organisms.

After this first check, the web servers (or stand-alone tools) are examined by myself and our team of in-depth testers. Our examinations include the function of the web server as well as the quality of documentation and sufficient and downloadable example data. We also check whether there is licence granting free access for every user, including companies, and if the web server can be used without registration. Starting with this issue we also require a cookie consent; because the number of third-party tracking cookies is steadily growing, we will tighten our cookie policy in the future.

The examination process for hundreds of web servers is cumbersome and I am therefore very happy to have two new testers in our team. Georges Schmartz and Viktoria Wagner, PhD students at the Universität des Saarlandes from Saarbrücken, Germany, join

- Adam Simpkin, who holds a PhD in X-ray crystallography and is at the University of Liverpool, UK
- Piotr Grabowski, who holds a PhD in computational systems biology and works for AstraZeneca in Cambridge, UK
- Lennard Ostendorf, who is a medical doctor and a PhD student at the Charité – Universitätsmedizin Berlin, Germany
- Sebastian Proft, who is a bioinformatician and PhD student in my group at the Berliner Institut für Gesundheitsforschung, Berlin, Germany

The authors of web servers that passed our tests were then invited to submit a manuscript—whose review(s) and revision(s) had to be completed in the short time frame before our internal publication deadline on 30 April.

The accepted manuscripts cover web servers (and one stand-alone tool) that support research activities in a wide range of areas, ranging from software aimed at the wet lab through structural biology to completely computational methods. This issue contains the following 89 manuscripts:

**Table:** 

ShortTitle	Text	URL
ADMETlab 2.0	Prediction of ADMET (absorption, distribution, metabolism, excretion, toxicity)	https://admetmesh.scbdd.com/
Amino Acid Interactions (INTAA) v2.0	Computation of energetics and conservation in biomolecular 3D structures	https://bioinfo.uochb.cas.cz/INTAA/
AnnotSV & knotAnnotSV	Annotation and interpretation of human structural variants	https://www.lbgi.fr/AnnotSV/
antiSMASH 6.0	Detection and characterisation of biosynthetic gene clusters in bacteria and fungi	https://antismash.secondarymetabolites.org/
Arena3Dweb	3D visualisation of multilayered networks	http://bib.fleming.gr/Arena3D
Aviator	Monitoring the availability of web services	https://www.ccb.uni-saarland.de/aviator
b2bTools	Predictions for protein biophysical features and their conservation	https://bio2byte.be/b2btools/
BENZ WS	Four-level Enzyme Commission (EC) number annotation	https://benzdb.biocomp.unibo.it/
BRIO	RNA sequence and structure motif scan	http://brio.bio.uniroma2.it/
catRAPID omics v2.0	Prediction of protein–RNA interactions	http://service.tartaglialab.com/page/catrapid_omics2_group
CeLaVi	Cell lineage visualisation	http://www.celavi.pro/
CheckMyBlob	Recognition and validation of ligands	https://checkmyblob.bioreproducibility.org/
CNVxplorer	Clinical interpretation of CNVs in rare disease patients	http://cnvxplorer.com/
CoffeeProt	Enrichment analysis for genetic data	http://www.coffeeprot.com/
CPA	Consensus pathway analysis	http://cpa.tinnguyen-lab.com/
CRISPRloci	Annotation of CRISPR-Cas systems	http://rna.informatik.uni-freiburg.de/trunk/CRISPRloci
DeepFun	Prediction of the effect of non-coding variants	https://bioinfo.uth.edu/deepfun/
DeepGOWeb	Protein function prediction on the semantic web	https://deepgo.cbrc.kaust.edu.sa/
DeepRefiner	Protein structure refinement	http://watson.cse.eng.auburn.edu/DeepRefiner/
DGLinker	Knowledge-graph prediction of disease-gene associations	https://dglinker.rosalind.kcl.ac.uk/
DoChaP	Comparison of protein domains between isoforms	https://dochap.bgu.ac.il/
DomainViz	Consensus domain distributions across groups of proteins	https://uhrigprotools.biology.ualberta.ca/domainviz
DrugComb	Drug sensitivity data repository	https://drugcomb.org/
EDGAR3.0	Comparative genomics and phylogenomics for microbial genomes	http://edgar3.computational.bio/
eSkip-Finder	Design of antisense oligonucleotides for exon skipping	https://eskip-finder.org/
Estimage	Computation of methylation age	http://estimage.iac.rm.cnr.it/
eVITTA	Transcriptome analysis	https://tau.cmmt.ubc.ca/eVITTA/
Expasy	The Swiss Bioinformatics Resource Portal	https://www.expasy.org/
G2PDeep	Deep-learning framework for phenotype prediction and discovery of genomic markers	http://g2pdeep.org/
GalaxyHeteromer	Protein heterodimer structure prediction	http://galaxy.seoklab.org/cgi-bin/submit.cgi?type=HETEROMER
GEPIA2021	Gene expression analysis	http://gepia2021.cancer-pku.cn/
GPCRsignal	G protein-coupled receptors and their effector proteins	https://gpcrsignal.biomodellab.eu/
Graphery	Tutorials for biological network algorithms	https://graphery.reedcompbio.org/
gutSMASH	Identification of primary metabolic gene clusters from the gut microbiota	https://gutsmash.bioinformatics.nl/
iNetModels 2.0	Visualisation and database of multi-omics data	https://inetmodels.com/
InterEvDock3	Protein docking	http://bioserv.rpbs.univ-paris-diderot.fr/services/InterEvDock3/
IPC 2.0	Prediction of isoelectric point and pKa dissociation constants	http://www.ipc2-isoelectric-point.org/
iTOL v5	Phylogenetic tree display and annotation	https://itol.embl.de/
IUPred3*	Prediction of intrinsically unstructured proteins	https://iupred3.elte.hu/
KEA3	Kinase enrichment analysis	https://maayanlab.cloud/kea3
KOBAS-i	Exploration of biological functions in gene set enrichment analysis	http://kobas.cbi.pku.edu.cn
LigAdvisor	Drug design	https://ligadvisor.unimore.it/
LipidSig	Lipidomics	http://chenglab.cmu.edu.tw/lipidsig/
LipidSuite	Lipidomics differential and enrichment analysis	http://suite.lipidr.org/
LitSuggest	Literature recommendation and curation	https://www.ncbi.nlm.nih.gov/research/litsuggest
LZerD	Pairwise and multiple protein-protein docking	https://lzerd.kiharalab.org/
Mechnetor	Exploration of the functional context of genetic variants	http://mechnetor.russelllab.org/
Mergeomics 2.0	Multi-omics and disease networks	http://mergeomics.research.idre.ucla.edu/
MetaboAnalyst 5.0	Metabolomics data analysis and interpretation	https://www.metaboanalyst.ca/
miRMaster 2.0	Multi-species non-coding RNA sequencing analysis	https://www.ccb.uni-saarland.de/mirmaster2
miRTargetLink2	miRNA target gene and target pathway networks	https://www.ccb.uni-saarland.de/mirtargetlink2
mmCSM-PPI	Effects of multiple point mutations on protein-protein interactions	http://biosig.unimelb.edu.au/mmcsm_ppi
ModFOLD8	Quality estimates for 3D protein models	https://www.reading.ac.uk/bioinf/ModFOLD/
Mol* Viewer	3D visualisation and analysis of large biomolecular structures	https://molstar.org/
MTR3D	MTR3D: Identification of regions under purifying selection in protein 3D structures	http://biosig.unimelb.edu.au/mtr3d/
MutationTaster2021	Prediction of the disease-causing potential of DNA variants	https://www.genecascade.org/MutationTaster2021/
MyCLADE	Multi-source domain annotation	http://www.lcqb.upmc.fr/myclade
ncFANs v2.0	Functional annotation of noncoding RNAs	http://ncfans.gene.ac/
NetGO 2.0	Protein function prediction	http://issubmission.sjtu.edu.cn/ng2/
OmicsAnalyst	Analysis of multi-omics data	https://www.omicsanalyst.ca/
OpenAnnotate	Annotation of chromatin accessibility of genomic regions	http://health.tsinghua.edu.cn/openannotate/
OxDNA.org	DNA and RNA nanostructures	https://oxdna.org/
PE-Designer & PE-Analyzer	CRISPR prime editing	http://www.rgenome.net/pe-designer/ and http://www.rgenome.net/pe-analyzer/
pegIT	Prime editing	https://pegit.giehmlab.dk/
PERCEPTRON	Proteoform identification pipeline	https://perceptron.lums.edu.pk/
pLannotate	Engineered plasmid annotation	http://plannotate.barricklab.org/
PlantDeepSEA	Prediction of the regulatory effects of genomic variants in plants	http://plantdeepsea.ncpgr.cn/
PLIP2021	Protein–ligand interaction profiler	https://plip-tool.biotec.tu-dresden.de/
PredictProtein	Prediction of protein structure and function	https://predictprotein.org/
Preselector.uni-jena.de	Identification of restriction enzymes for preselection reactions	https://preselector.uni-jena.de/
ProLint	Lipid–protein interactions	http://www.prolint.ca/
ProteinLens	Analysis of allosteric signalling on atomistic graphs of biomolecules	http://www.proteinlens.io/
ProteinTools	Protein structure analysis toolkit	https://proteintools.uni-bayreuth.de/
Proteo3Dnet	Analysis of mass spectrometry interactomics experiments	http://bioserv.rpbs.univ-paris-diderot.fr/services/Proteo3Dnet/
ProteoSign v2	Statistical analysis of differential proteomics	http://bioinformatics.med.uoc.gr/ProteoSign
ProteoVision	Visualization of ribosomal proteins	http://proteovision.chemistry.gatech.edu/
ReFOLD3	Refinement of 3D protein models	https://www.reading.ac.uk/bioinf/ReFOLD/
RunBioSimulations	Platform for computational modelling frameworks, algorithms, formats	https://run.biosimulations.org/
snpXplorer	Annotation and exploration of human SNPs	https://snpxplorer.eu.ngrok.io/
SynLeGG	Analysis of multi-omics data for discovery of cancer ‘achilles heels'	http://www.overton-lab.uk/synlegg
The COVID-19 Data Portal	COVID-19 data repository	
The Dockstore	Platform for sharing computational protocols	https://dockstore.org/
Thunor	Analysis of dose-response datasets	http://www.thunor.net/
TIMEOR	Uncovering temporal regulatory mechanisms from multi-omics data	https://timeor.brown.edu/
TISIGNER.com	Improving recombinant protein production	https://tisigner.com/
Trips-Viz	Ribosome profiling	https://trips.ucc.ie/
Vaxign2	Vaccine design software	http://www.violinet.org/vaxign2
VirtualTaste	Prediction of organoleptic properties of chemical compounds	http://virtualtaste.charite.de/VirtualTaste/
Voronoia 4-ever	Calculation of internal packing densities in macromolecules	https://proteinformatics.uni-leipzig.de/voronoia/

In the editorial describing the 2020 Web Server Issue I had highlighted a survey on the availability of web servers by Andreas Keller's group (Universität des Saarlandes, Saarbrücken, Germany). In the meantime, a new web service has emerged from this endeavour: with Aviator (https://ccb-compute2.cs.uni-saarland.de/aviator/), the state (or fate) of almost 10 000 scientific web servers can be studied – and while I am proud that *Nucleic Acids Research* has published more of them than any other journal, I notice with unease that >5% of the web servers published in 2020 in any journal are permanently offline. As we state in our guide to authors, we expect any web server published in the Web Server Issue to be maintained for at least 5 years. In the future, I will use Aviator to reject proposals from authors who abandon their web servers soon after publication.

Further planned changes for the 2022 Web Server Issue are:

- We will tighten our cookie policy. As with the ‘no login’ requirement, we do not want researchers to be tracked. We will allow third-party cookies only when they are needed for the function of the website.
- We will lift our ‘no login’ requirement in favour of security **if and only if** sensitive non-public human data such as genotypes from WES, WGS, WTS or SNP arrays is transmitted. Registration must be possible without any identification (name or email address).
- Mandatory use of https if web servers handle sensitive human data.

With this outlook, I want to close the editorial—I hope you will find many of the new web servers exciting and helpful.

I want to thank our in-depth testers and the staff at Oxford University Press for their help, especially Martine Bernardes Silva and Karen Coldwell from our editorial office—without them, this issue would not have been possible. My thanks also go to the hundreds of referees who reviewed the submitted manuscripts in a very short time-frame and to Dan Rigden from the Executive Editor team for help with handling some proposals I had a conflict of interest with.

Please check https://academic.oup.com/nar/pages/submission_webserver for any changes of deadline or requirements by November if you want to submit to the 2022 Web Server Issue. As the vast majority of proposals are uploaded in the last 2 days before the deadline, I want to encourage you to submit as soon as possible! This balances the burden on our in-depth reviewers, the referees, and it gives you more time to revise your software and manuscripts.

